# Quality and reliability of semaglutide content on different Chinese short video platforms: a study focusing on side effects

**DOI:** 10.3389/fpubh.2026.1750738

**Published:** 2026-02-24

**Authors:** Jiayuan Song, Xingchen He, Zhenhai Cui, Ye Sun, Yanlin Liu, Han Yu, Aimin Li, Meiying Jin

**Affiliations:** 1 College of Integrative Medicine, Changchun University of Chinese Medicine, Changchun, China; 2 The Affiliated Guangzhou Hospital of TCM of Guangzhou University of Chinese Medicine, Guangzhou, China; 3 Department of Orthopedics, The Third Affiliated Hospital of Changchun University of Traditional Chinese Medicine, Changchun, China; 4 Department of Endocrine Metabolism and Spleen and Stomach, The Third Affiliated Hospital of Changchun University of Traditional Chinese Medicine, Changchun, China

**Keywords:** content characteristics, creator identity, information quality, semaglutide, short-video platforms, side effects

## Abstract

**Objective:**

This study aims to evaluate the quality, reliability, and content characteristics of semaglutide-related short videos on three major Chinese short-video platforms (Bilibili, TikTok, and Rednote), with a focus on exploring differences in the presentation of side effects across different platforms and uploader types. Rather than examining specific clinical indications or dosing regimens, this study focuses on how semaglutide-related information is presented to the general public in real-world short-video contexts. It seeks to clarify the impact of platform ecology and creator identity on drug information dissemination, thereby providing a basis for optimizing the quality of online health information and conducting targeted public health communication.

**Methods:**

A cross-sectional content analysis was adopted. Semaglutide-related videos were retrieved from three major Chinese short-video platforms using the Chinese keyword “司美格鲁肽” (the standard Mandarin term for “semaglutide”). This exact search term was used to simulate real-world user behavior and ensure reproducibility across platforms. Because most videos did not specify approved indications (type 2 diabetes vs. obesity) or dosage levels, analyses were conducted without stratification by clinical dose or indication. After rigorous screening, eligible videos were assessed for quality and reliability using the modified DISCERN scale and Global Quality Score (GQS). Video content was categorized into five thematic dimensions, and mentions of nine predefined side effects were systematically recorded. A dual-perspective analytical approach—comparing the full sample and a side effect–focused subsample—was employed to examine platform-based differences in adverse event portrayal.

**Results:**

A total of 607 semaglutide-related videos were included (Bilibili: 309, TikTok: 153, Rednote: 145). The results showed significant differences in content quality (M-DISCERN and GQS scores), video duration, and user engagement metrics across platforms (*p* < 0.05). There were statistically significant differences in content distribution among platforms (*p* < 0.05): Bilibili focused on Policy News and Events, Personal Experience Sharing, and Commercial Promotion; Rednote centered on Medication Education and Medical Advice/Medication Guidance; TikTok mainly focused on Policy News and Events. Regarding side effects, the distribution of gastrointestinal and hepatobiliary side effects among the three platforms was statistically significant (*p* < 0.05), with TikTok having the highest mention rate. These side effect narratives were typically presented without reference to specific dosing levels or approved indications. The dual-perspective analysis further revealed systematic differences in side effect portrayal, with TikTok conferring greater visibility for adverse-event narratives. In terms of content quality, the three platforms showed a gradient difference of “Rednote > TikTok > Bilibili” (*p* < 0.001).

**Conclusion:**

The quality and reliability of semaglutide-related short videos are significantly influenced by platform ecology. Publicly accessible drug information on short-video platforms often lacks clear differentiation between approved indications and dosage regimens, contributing to generalized interpretations of drug risks. The drug information accessible to the public exhibits structural biases, with side effect narratives demonstrating a clear “platformization” pattern. The dual-perspective analysis revealed that inter-platform differences in the portrayal of specific adverse events (e.g., gastrointestinal and hepatobiliary effects) were more discernible in the side effect-focused subsample. Future interventions should be tailored to platform-specific features to enhance the completeness and scientific rigor of online pharmaceutical information.

## 1 Introduction

Semaglutide, a glucagon-like peptide-1 (GLP-1) receptor agonist, has demonstrated significant weight-loss and cardiovascular benefits in clinical trials, while also sparking widespread public discussion and media attention worldwide (1). Its popularity has been largely driven by social media dissemination, resulting in soaring market demand and global shortages (2). In China, semaglutide quickly became a focus of public debate following its approval for weight-loss indications. On social media platforms, medical professionals display considerable variation in their portrayals of its efficacy and risks, with major perspectives including framing it as a “medical breakthrough,” cautioning against its misuse as a “non-slimming shortcut,” and expressing nuanced attitudes toward patient use (3). These discussions reflect the intertwining of the medicalization of obesity and discourses of personal responsibility in public health communication, while also revealing the communicative dilemma faced by Chinese medical disseminators in balancing promotion and risk warning.

Meanwhile, the enormous popularity of semaglutide has brought challenges concerning information quality and safety risks. In addition to legitimate medications, unregulated synthetic versions have appeared on the market, whose uncertain dosages and potential side effects have raised public concern (2). Against this backdrop, social media has become a primary channel through which the public obtains drug-related information, yet issues of content quality and reliability have become increasingly prominent. At the international level, Campos-Rivera et al. analyzed Spanish-language TikTok videos and found that semaglutide-related content had an average DISCERN score of only 29.8 out of 75, with 71% of videos failing to mention any side effects. Instead, these videos reinforced social notions that attribute obesity to individual responsibility, revealing the potential public health risks posed by the commercial motivations behind the “pharmaceuticalization of obesity” (4). Similarly, Yeung et al. examined 189 English-language YouTube videos and found that both short and long formats generally lacked in-depth discussion of critical issues such as long-term safety, effects of drug half-life, and counterfeit drug risks. Even videos produced by verified medical sources failed to adequately address potential serious adverse events of regulatory and public health interest, such as pancreatitis and gallbladder disease, for which causal associations with semaglutide remain under investigation (5). A cross-platform comparative study by Propfe and Seifert further revealed the significant influence of platform ecology on information quality: YouTube, with a higher proportion of medical professional creators (48%), emerged as the most reliable platform. In contrast, Instagram and TikTok were dominated by non-professional users, resulting in the lowest information quality, with key risk information such as gastrointestinal side effects entirely absent on Instagram (6). The study’s information scoring system showed that average scores across platforms were generally low (YouTube mean 1.38, Instagram mean 0.22), highlighting the systemic shortcomings of social media in disseminating critical drug information.

In the Chinese context, Huang conducted a content analysis of medical professionals on the TikTok platform, revealing a complex narrative tension: semaglutide is simultaneously portrayed as a “medical breakthrough” and cautiously framed as “not a shortcut to weight loss,” demonstrating the interweaving of discourses of medical authority and self-responsibility. This narrative framework not only reflects the communicative dilemma faced by professional disseminators in balancing drug promotion and risk warning, but also exposes the potential influence of “fat phobia” and the moralization of the body in Chinese sociocultural contexts on health communication (3). A synthesis of existing studies shows that common issues in semaglutide-related content on social media include uneven information quality, selective presentation of risk information, and the infiltration of commercial logic into health communication. However, current research mainly focuses on Western mainstream platforms, while systematic evaluations are lacking regarding the patterns of drug information dissemination—especially the reliability and communicative characteristics of side-effect information—on uniquely Chinese short-video ecosystems such as TikTok, Rednote, and Bilibili. Given the significant role these platforms play in Chinese public access to health information, conducting relevant research holds considerable theoretical and practical significance.

Therefore, this study aims to conduct a cross-platform comparative analysis of semaglutide-related short videos on three major Chinese platforms—Bilibili, TikTok, and Rednote. The primary objective is to evaluate and compare the overall quality and reliability of these videos, with a specific focus on how side effects are presented across different platforms and uploader types. The secondary objectives include: (1) characterizing the thematic content distribution of semaglutide-related videos; (2) identifying platform- and creator-specific patterns in the reporting of specific side effect categories (e.g., gastrointestinal, hepatobiliary, ocular); and (3) examining the association between video quality metrics (as measured by modified DISCERN and GQS) and user engagement indicators. By clarifying these dimensions, this study seeks to inform evidence-based strategies for tailoring public health communication to the unique affordances of each platform.

## 2 Method

### 2.1 Search strategy and data screening

**Figure 1:**
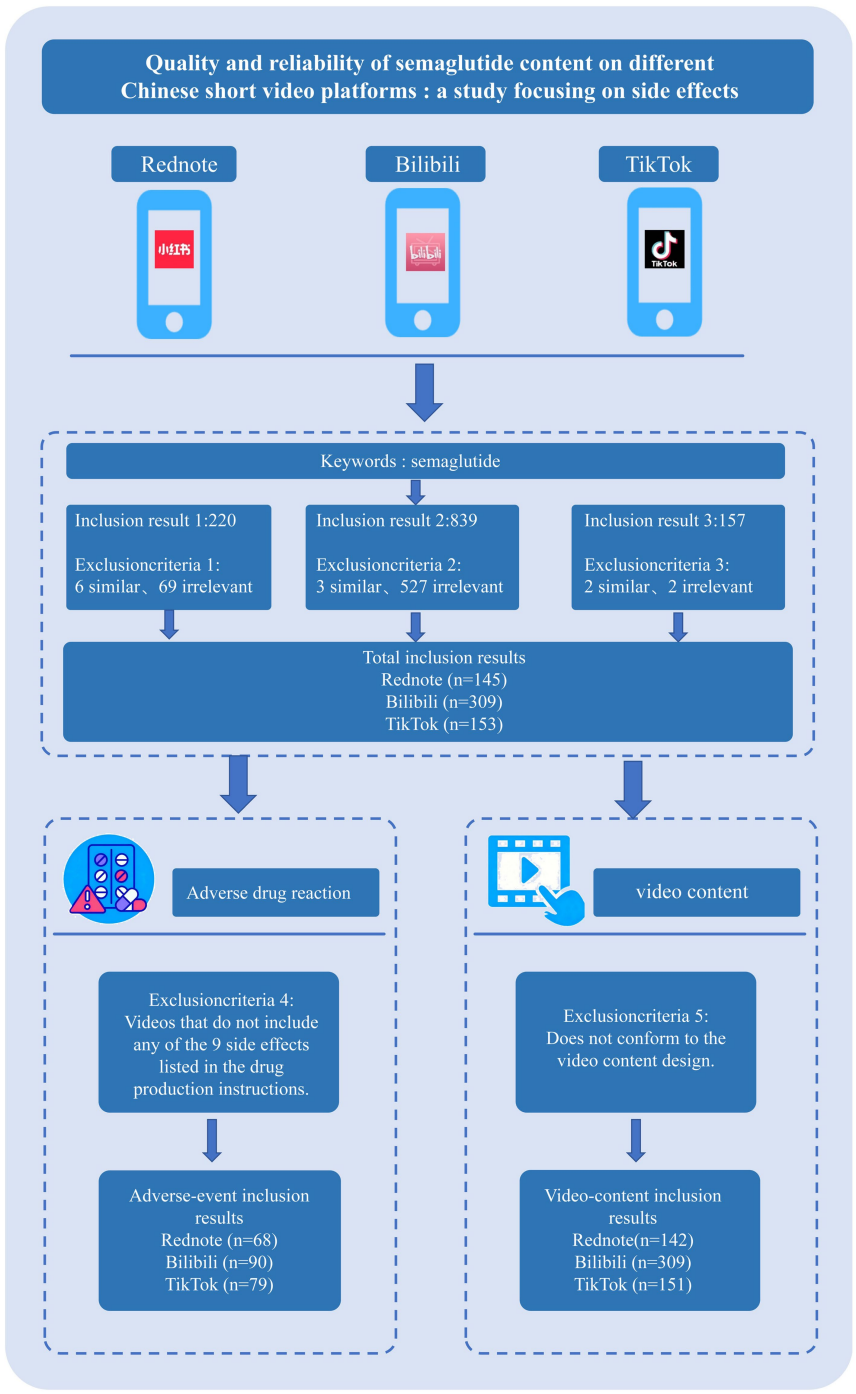
Flowchart of videos related to semaglutide included in this study.

In this cross-sectional analysis, researcher Jiayuan Song retrieved semaglutide-related videos from TikTok, Bilibili, and Rednote on 21 September 2025, covering the entire period from each platform’s inception to that date. Bilibili is characterized by medium- to long-form science-popularization videos and a young, knowledge-oriented user base (7); TikTok relies on algorithmic recommendations, offers strong interactivity, and reaches a broad audience (8); Rednote features a community-validated “note” ecosystem with high demand for health and lifestyle information (9–13). Collectively, the three platforms encompass diverse user demographics and content ecosystems, thereby ensuring the representativeness of the sample. The Chinese keyword “司美格鲁肽” (corresponding to “semaglutide” in English) was used for the search. Notably, videos were not screened or stratified according to approved clinical indications (e.g., type 2 diabetes versus obesity management) or dosage regimens, because the majority of videos did not explicitly specify these details. Instead, the present study focused on all publicly accessible semaglutide-related content encountered by general users on short-video platforms. To guarantee data accuracy, we conducted comprehensive inclusion; exclusion criteria comprised duplicate content and irrelevant topics (Figure 1). After applying these criteria, 607 eligible short videos were retained: 309 from Bilibili (50.91%), 153 from TikTok (25.21%), and 145 from Rednote (23.89%).

### 2.2 Video data collation

For each included video, the extracted dataset comprised title, uploader identity, duration, number of likes, comments, favorites, shares, and days since upload. The variables analyzed were duration, likes, comments, shares, favorites, video content, and the nine known adverse effects of semaglutide. Mentions of adverse effects were recorded regardless of whether videos specified a particular indication or dosage, reflecting how drug risks are communicated in public-facing short-video environments rather than within a formal clinical context. Uploaders were dichotomized into medical professionals and non-medical professionals. Medical professionals encompassed professional individuals (licensed physicians, non-licensed physicians, and other health-care personnel) and professional institutions, whereas non-medical professionals comprised non-professional individuals (patients and science bloggers) and non-professional institutions (news agencies and non-profit organizations). Based on actual content, videos were taxonomized into five categories: Medication Education, Policy News and Events, Medical Advice / Medication Guidance, Personal Experience Sharing, and Commercial Promotion. The adverse-effect profile of semaglutide was referenced from the prescribing information of Rybelsus (oral semaglutide) and Ozempic (injectable semaglutide), with details provided in Supplementary Tables 1, 2 (5, 14–17). These references were used solely to define and standardize adverse-effect categories for content coding, rather than to establish dose-specific or indication-specific risk attribution.

### 2.3 Video quality assessment

Each video was independently reviewed by two raters using the Global Quality Score (GQS) and the modified DISCERN (DIS) instrument. The GQS is a validated instrument for appraising video quality across five dimensions. The GQS uses a 5-point scale (1 = poor, 5 = excellent) derived from a holistic appraisal of video content and presentation quality (18, 19). Quality assessment focused on clarity, balance, and reliability of information as presented to the general public, rather than on the accuracy of dose-specific clinical recommendations. Content accuracy was appraised against Chinese and international myopia-control guidelines (20–22) and recent systematic reviews or meta-analyses on myopia management (23, 24). The modified DIS tool comprises five yes/no items scored 1 or 0, giving a range of 0–5 points. Discrepancies between raters were resolved through discussion with a senior reviewer, as detailed in Supplementary Tables 3, 4. To minimize platform-algorithm bias, searches were performed with new smartphones and newly registered accounts. Eligible video URLs were saved and distributed to reviewers via a secure shared document. Every assessor accessed and scored the videos in private-browsing mode to prevent recommendation-algorithm interference.

### 2.4 Statistical analysis

Statistical analysis and visualization were performed using IBM SPSS Statistics 26.0, and R 4.3.0. Comparisons between two groups were conducted using the independent-samples t-test. Non-normally distributed data are presented as median (interquartile range, M [Q1, Q3]). For analyses related to side effects and video content, R 4.3.0 was used. All analyses treated adverse-effect mentions as content-level variables without stratification by clinical indication or dosage, consistent with the descriptive and communication-focused nature of this study. Categorical data are expressed as n (%), and group comparisons were performed using the chi-square test or Fisher’s exact test. A *p*-value < 0.05 was considered statistically significant.

## 3 Results

### 3.1 Basic characteristics

A total of 607 videos were included in the analysis. When stratified by platform, Platform 1, 2, and 3 (Bilibili, TikTok, and Rednote) contributed 309 (50.91%), 153 (25.21%), and 145 (23.89%) videos, respectively. Inter-platform differences in M-DISCERN score, GQS score, video duration, likes, comments, shares, and favorites were all statistically significant (*p* < 0.05; Table 1).

**Table 1:** General information, quality, and reliability scores of semaglutide-related videos on different Chinese short-video platforms.

Variables	Total (*n* = 607)	Bilibili (*n* = 309)	TikTok (*n* = 153)	Rednote (*n* = 145)	Statistic	*p*
M Discern Score, Mean ± SD	1.55 ± 0.86	1.46 ± 0.88	1.59 ± 0.90	1.68 ± 0.73	*F* = 3.34	**0.036**
GQS score, Mean ± SD	2.22 ± 0.92	1.93 ± 0.76	2.74 ± 1.11	2.30 ± 0.73	*F* = 47.16	**<0.001**
Duration, M (Q₁, Q₃)	124.00 (62.50, 210.50)	147.00 (84.00,284.00)	107.00 (60.00,161.00)	111.00 (50.00,156.00)	χ^2^ = 29.56#	**<0.001**
Like, M (Q₁, Q₃)	44.00 (8.50, 567.00)	12.00 (4.00,45.00)	2300.00 (292.00,9480.00)	59.00 (12.00,263.00)	χ^2^ = 252.23#	**<0.001**
Comment, M (Q₁, Q₃)	17.00 (2.00, 154.00)	8.00 (1.00,39.00)	478.00 (60.00,1662.00)	7.00 (1.00,60.00)	χ^2^ = 171.45#	**<0.001**
Share, M (Q₁, Q₃)	15.00 (2.00, 335.50)	4.00 (1.00,20.00)	1510.00 (196.00,9040.00)	35.00 (4.00,177.00)	χ^2^ = 242.62#	**<0.001**
Collect, M (Q₁, Q₃)	19.00 (3.00, 204.00)	6.00 (1.00,24.00)	863.00 (71.00,4511.00)	25.00 (4.00,129.00)	χ^2^ = 207.44#	**<0.001**

F, ANOVA, #, Kruskal-waills test. SD, standard deviation; M, Median; Q₁, 1st Quartile; Q₃, 3st Quartile.Bold values indicate statistical significance at *p* < 0.05.

**Figure 2:**
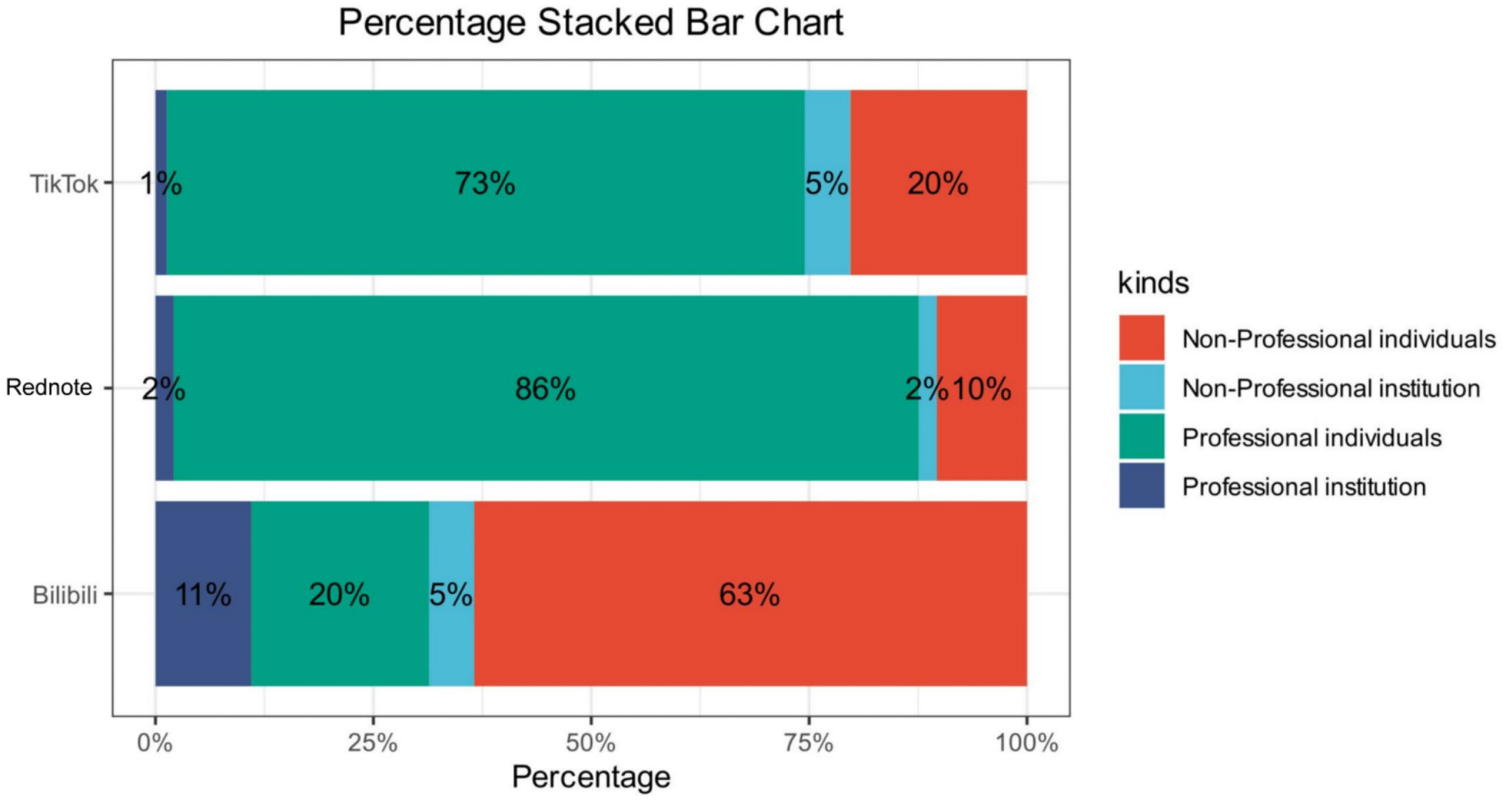
Numbers of video uploaders regarding Semaglutide across different short-video platforms.

**Figure 3:**
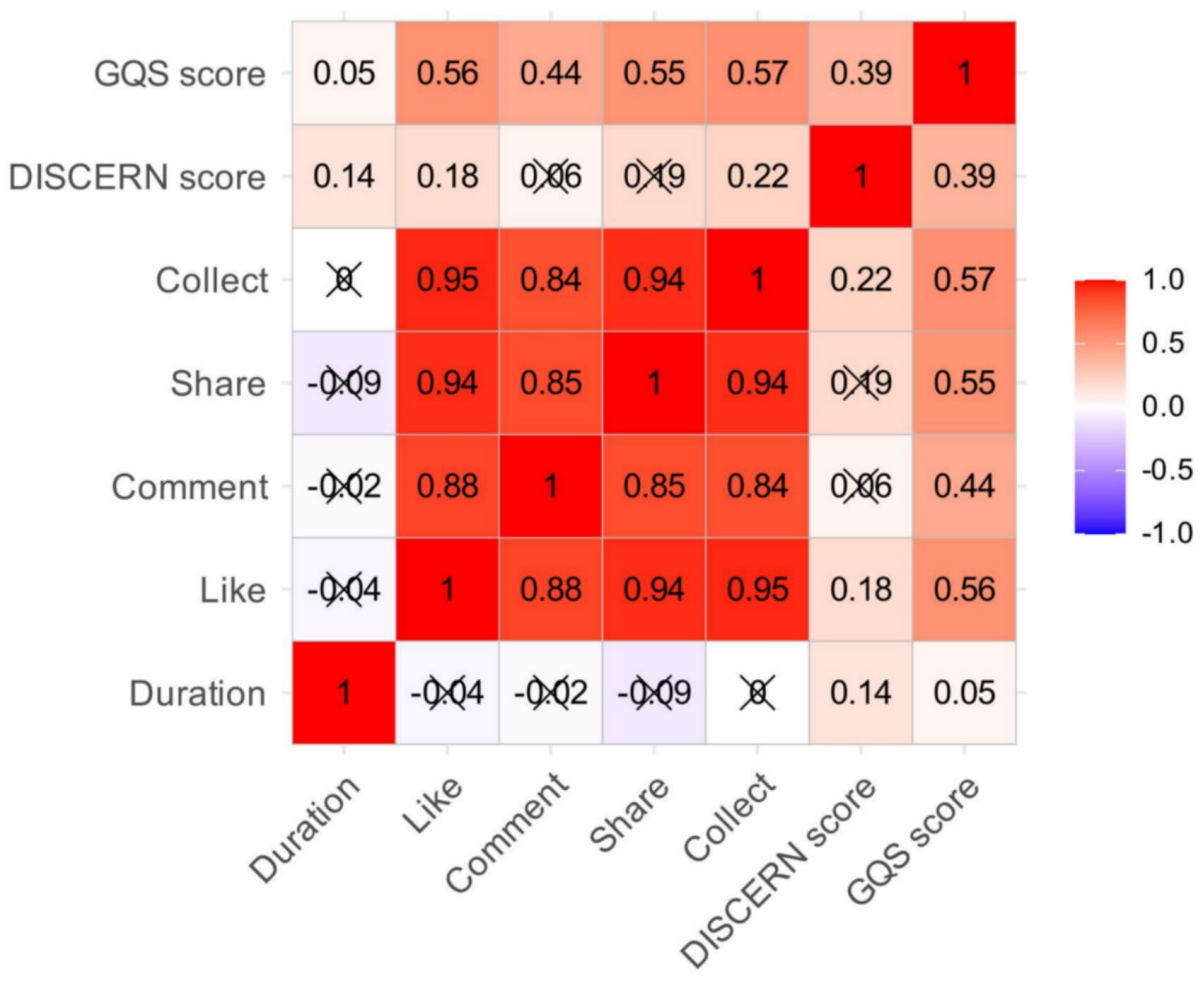
Correlation analysis of general characteristics: A heatmap revealing synergistic user engagement behaviors.

Second, when classified by individual creator identity, a stacked bar chart clearly illustrates the differences in uploaders across the three platforms: TikTok and Rednote are dominated by non-professional institutions, whereas Bilibili is primarily composed of non-professional individuals (Figure 2). A heat map visually presents the intercorrelations among general variables of semaglutide-related videos, with the strongest correlation coefficients between favorites and likes (R = 0.95) and between shares and likes (R = 0.94), indicating a strong “synergistic effect” or “behavioral consistency” in user interactions (Figure 3). In contrast, video publication duration showed no significant correlation with any of these metrics, suggesting that for high-value, highly interactive, and frequently searched health content such as semaglutide, content quality and user interest determine long-term performance, whereas publication timing has virtually no influence on ultimate dissemination outcomes.

When stratified by creator identity, the sample comprised 242 non-professional individuals (44.73%), 27 non-professional institutions (4.45%), 299 professional individuals (55.27%), and 39 professional institutions (6.43%). Across the four creator categories, global differences in video quality (M-DISCERN and GQS scores) and all interaction metrics—duration, likes, comments, shares, and favorites—were statistically significant (*p* < 0.05; Table 2). Subgroup analysis of individual creators (Supplementary Table 5) revealed that professional individuals achieved significantly higher M-DISCERN (1.80 ± 0.81) and GQS (2.55 ± 0.87) scores than non-professional individuals (1.21 ± 0.77 and 1.78 ± 0.77, respectively; *p* < 0.001 for both). Moreover, professional individuals garnered markedly more likes, comments, shares, and favorites, while their videos were shorter, indicating that concise, high-quality content from credentialed creators drives greater user engagement (Supplementary Table 5). Among institutional creators (Supplementary Table 6), no statistically significant differences were observed between professional and non-professional institutions in core quality metrics or video duration. However, non-professional institutions outperformed professional institutions in likes, comments, and shares (all *p* < 0.05), with only favorites showing no significant difference (*p* = 0.328), suggesting that branding and dissemination strategies may exert a stronger influence on interactive visibility than intrinsic content quality.

**Table 2:** Characteristics, quality, and reliability of semaglutide-related videos by different uploaders on different Chinese short-video platforms.

Variables	Total (*n* = 607)	Non-professional individuals (*n* = 242)	Non-professional institutions (*n* = 27)	Specialized individual (*n* = 299)	Professional institutions (*n* = 39)	Statistic	*p*
m DISCERN score, Mean ± SD	1.55 ± 0.86	1.21 ± 0.77	1.37 ± 0.93	1.80 ± 0.81	1.82 ± 0.91	*F* = 25.96	**<0.001**
GQS score, Mean ± SD	2.22 ± 0.92	1.78 ± 0.77	2.63 ± 0.93	2.55 ± 0.87	2.18 ± 0.97	*F* = 39.81	**<0.001**
Duration, M (Q₁, Q₃)	124.00 (62.50, 210.50)	148.50 (99.00,276.25)	102.00 (40.50,217.00)	110.00 (56.00,158.00)	148.00 (60.00,310.50)	χ^2^ = 29.48#	**<0.001**
Like, M (Q₁, Q₃)	44.00 (8.50, 567.00)	14.50 (4.00,71.00)	360.00 (25.00,2192.00)	144.00 (22.00,1778.00)	14.00 (4.00,94.50)	χ^2^ = 86.90#	**<0.001**
Comment, M (Q₁, Q₃)	17.00 (2.00, 154.00)	10.00 (1.25,58.75)	238.00 (6.00,656.50)	25.00 (2.50,282.00)	6.00 (0.50,46.00)	χ^2^ = 19.60#	**<0.001**
Share, M (Q₁, Q₃)	15.00 (2.00, 335.50)	4.00 (0.00,33.50)	72.00 (6.50,3293.00)	70.00 (6.50,1259.50)	7.00 (2.00,69.50)	χ^2^ = 91.24#	**<0.001**
Collect, M (Q₁, Q₃)	19.00 (3.00, 204.00)	6.00 (1.00,31.00)	127.00 (4.50,378.00)	53.00 (7.00,805.50)	11.00 (4.00,38.00)	χ^2^ = 79.01#	**<0.001**

F, ANOVA, #, Kruskal-waills test. SD, standard deviation; M, Median; Q₁, 1st Quartile; Q₃, 3st Quartile.Bold values indicate statistical significance at *p* < 0.05.

**Figure 4:**
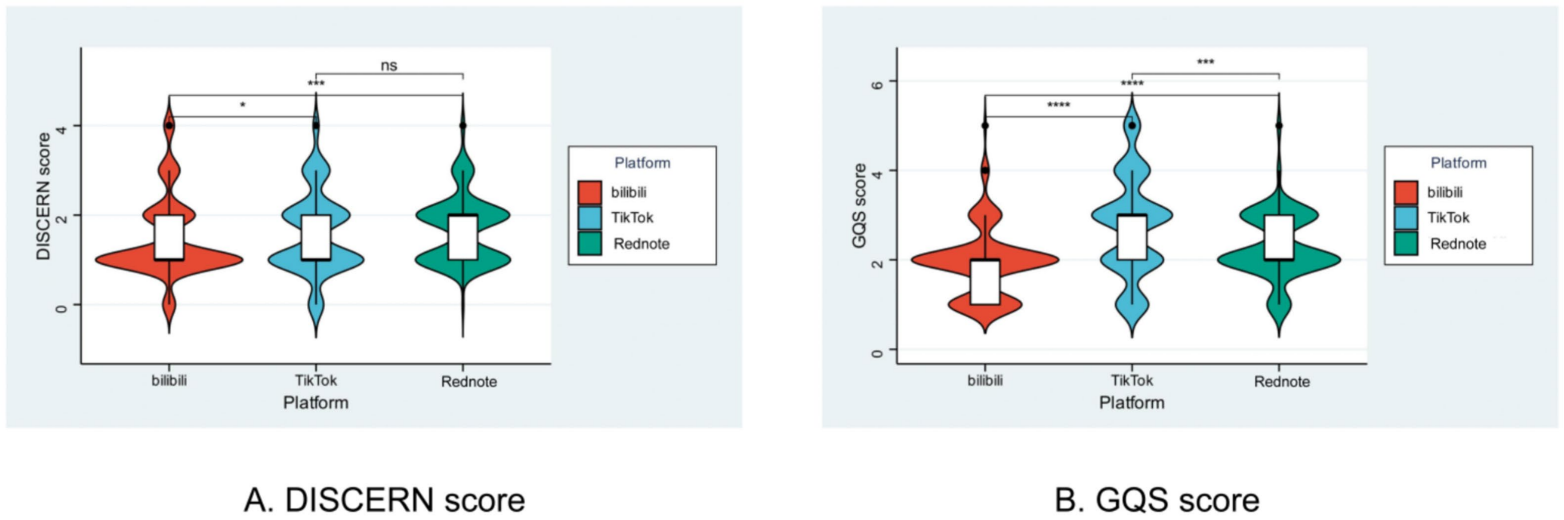
Distribution of GQS and DISCERN scores for semaglutide-related short videos across different platforms, visualized by violin plots. **(A)** DISCERN score. **(B)** GQS score. *p < 0.05.

**Figure 5:**
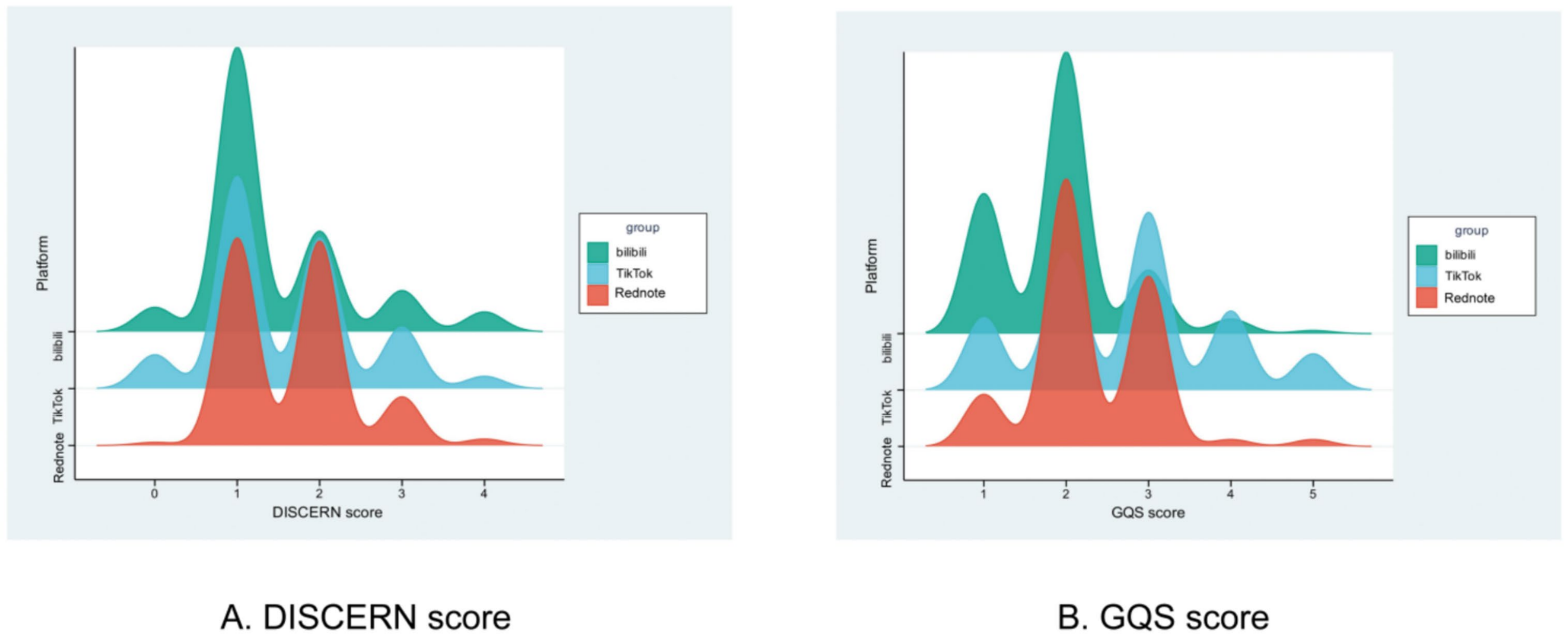
Distribution of GQS and DISCERN scores for semaglutide-related short videos across different platforms, visualized by the ridge plot. **(A)** DISCERN score. **(B)** GQS score. *p < 0.05.

To appraise platform-specific content quality regarding semaglutide, we applied both the DISCERN scale and the Global Quality Scale (GQS), with distributions and disparities visualized through violin and ridge plots. An overall “Rednote > TikTok > Bilibili” gradient in short-video quality was consistently observed. Violin plots revealed that Bilibili’s mDISCERN scores were significantly lower than those of the other two platforms, whereas GQS scores differed pairwise among all three platforms (*p* < 0.001), indicating highly significant quality gaps. Ridge plots further detailed that Bilibili peaked at DISCERN = 1 and GQS = 1–2, TikTok shifted to DISCERN = 1–2 and GQS = 2–3, while Rednote centered DISCERN at 2 and exhibited the highest proportion of high GQS scores (≥3; Figures 4, 5). These findings demonstrate an ascending “professional–science” density driven by platform ecology: Rednote’s tradition of in-depth, community-validated notes and its active female weight-management community foster high-evidence, high-information videos. TikTok’s massive algorithmic traffic is offset by an entertainment-oriented design that reduces depth, placing its quality in the middle. Bilibili’s former advantage in medium- to long-form science communication has been diluted by the shift to short videos, and its relatively small health-audience niche allows low-scoring content to proliferate. Creators seeking to elevate semaglutide content should prioritize Rednote’s “strong evidence + contextualized narrative” model and avoid Bilibili-style pure information reposting.

### 3.2 Video content and adverse-effect coverage

**Figure 6:**
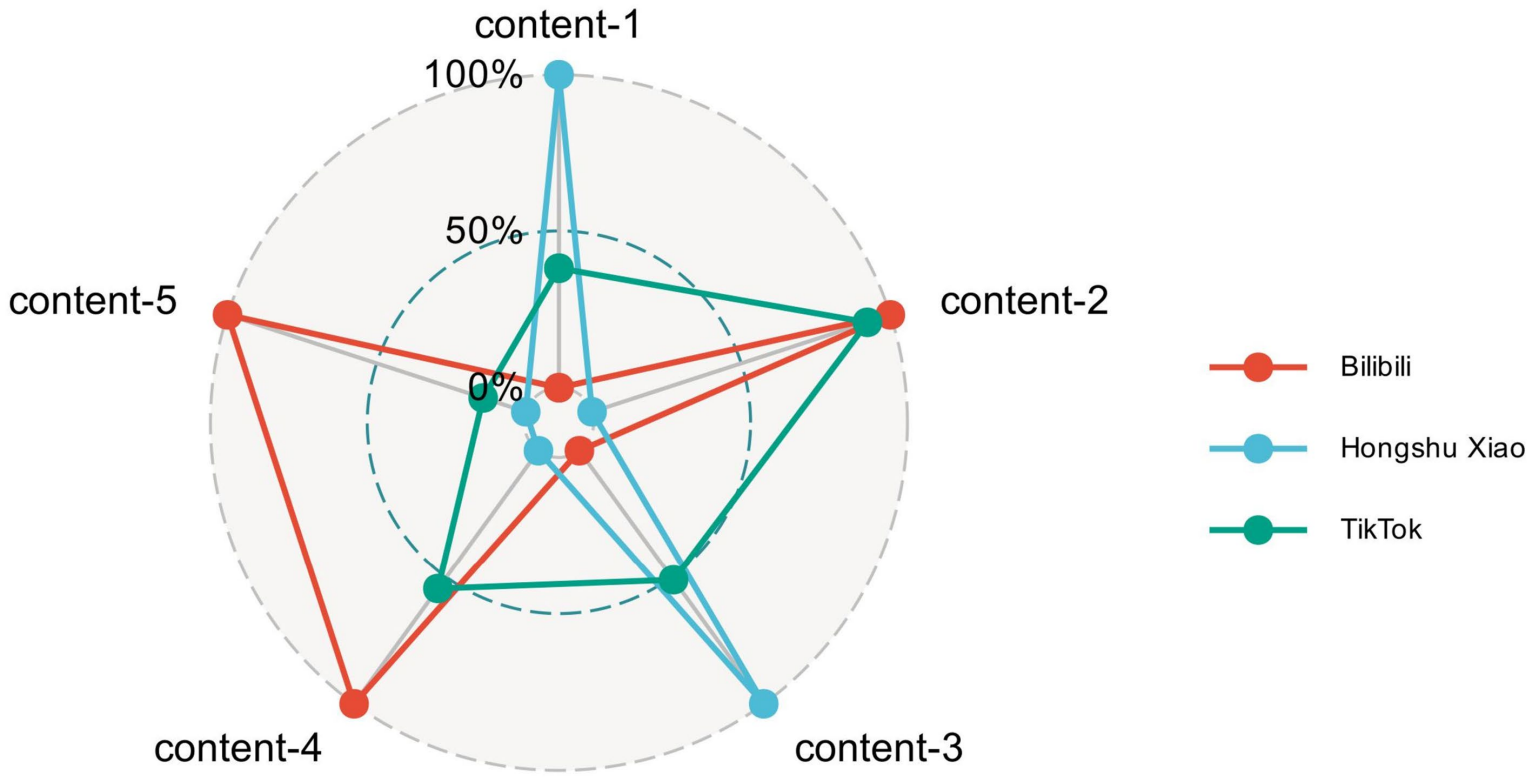
Differences in video content distribution across short-video platforms, represented by a radar chart. Video content-1: Medication education; video content-2: policy news and events; video content-3: medical advice/medication guidance; video content-4: personal experience sharing; video content-5: commercial promotion.

A radar chart (Figure 6) visualizes the distribution of videos across five content dimensions for each platform. Bilibili concentrates on dimensions 2, 4, and 5 (policy news/events, personal-experience sharing, and commercial promotion). Rednote emphasizes dimensions 1 and 3 (medication education and professional medical advice/medication guidance). TikTok is dominated by dimension 2 (policy news/events). Comparative analyses revealed statistically significant differences in content distribution among platforms (*p* < 0.05; Supplementary Tables 7).

Given that a substantial proportion of videos (61%) did not mention any side effects, distinguishing between the overall information landscape and the side-effect-specific narrative is crucial for a precise understanding of platform-specific communication characteristics. Therefore, we conducted a dual-perspective analysis of (i) the full sample (*n* = 607 videos, covering all eligible clips) and (ii) the side-effect-focused subsample (*n* = 237 videos that explicitly mention adverse events; Table 3). This approach uncovered systematic differences in how semaglutide-related adverse effects are portrayed on Chinese short-video platforms. Cross-validation between the two samples strengthened the robustness of our key findings: in both sets TikTok showed a significantly higher proportion of mentions of gastrointestinal and hepatobiliary side-effects than the other platforms (full sample: both *p* < 0.001 and *p* = 0.002; core sample: both *p* < 0.001 and *p* = 0.035), indicating that TikTok confers greater visibility and a stronger propensity for disseminating adverse-event narratives. More importantly, by restricting the analysis to videos that actually discuss side-effects, the core sample revealed fine-grained patterns that were masked in the full sample. For example, “abnormal laboratory findings” exhibited a significant between-platform difference in the core sample (*p* = 0.036) but not in the full sample (*p* = 0.613). This suggests that, once videos omitting any mention of side-effects are excluded, genuine inter-platform disparities emerge in the communication of adverse events that require professional medical interpretation.

**Table 3:** Comparison of semaglutide-related adverse events across Chinese short-video platforms: full sample vs. side effect-focused subsample analysis.

Variables	Comprehensive collation perspective						Side effect-focused perspective					
	Total (*n* = 607)	Bilibili (*n* = 309)	Rednote (*n* = 145)	TikTok (*n* = 153)	Statistic	*p*	Total (*n* = 237)	Bilibili (*n* = 90)	Rednote (*n* = 79)	TikTok (*n* = 68)	Statistic	*p*
Abnormal lab findings, n(%)					χ^2^ = 0.98	0.611					χ^2^ = 6.67	**0.036**
No	557 (91.76)	283 (91.59)	131 (90.34)	143 (93.46)			187 (78.90)	64 (71.11)	69 (87.34)	54 (79.41)		
Yes	50 (8.24)	26 (8.41)	14 (9.66)	10 (6.54)			50 (21.10)	26 (28.89)	10 (12.66)	14 (20.59)		
Gastrointestinal side effects, n(%)					χ^2^ = 32.44	**<0.001**					χ^2^ = 13.69	**0.001**
No	435 (71.66)	248 (80.26)	103 (71.03)	84 (54.90)			65 (27.43)	29 (32.22)	10 (12.66)	26 (38.24)		
Yes	172 (28.34)	61 (19.74)	42 (28.97)	69 (45.10)			172 (72.57)	61 (67.78)	69 (87.34)	42 (61.76)		
Metabolic side effects, n(%)					χ^2^ = 0.78	0.676					χ^2^ = 4.12	0.128
No	523 (86.16)	270 (87.38)	123 (84.83)	130 (84.97)			153 (64.56)	51 (56.67)	56 (70.89)	46 (67.65)		
Yes	84 (13.84)	39 (12.62)	22 (15.17)	23 (15.03)			84 (35.44)	39 (43.33)	23 (29.11)	22 (32.35)		
Cardiac side effects, n(%)					-	0.447					-	0.529
No	598 (98.52)	305 (98.71)	144 (99.31)	149 (97.39)			228 (96.20)	86 (95.56)	75 (94.94)	67 (98.53)		
Yes	9 (1.48)	4 (1.29)	1 (0.69)	4 (2.61)			9 (3.80)	4 (4.44)	4 (5.06)	1 (1.47)		
Hepatobiliary side effects, n(%)					-	**0.005**					χ^2^ = 6.71	**0.035**
No	591 (97.36)	305 (98.71)	143 (98.62)	143 (93.46)			221 (93.25)	86 (95.56)	69 (87.34)	66 (97.06)		
Yes	16 (2.64)	4 (1.29)	2 (1.38)	10 (6.54)			16 (6.75)	4 (4.44)	10 (12.66)	2 (2.94)		
Neurological side effects, n(%)					χ^2^ = 1.09	0.581					χ^2^ = 0.22	0.895
No	583 (96.05)	299 (96.76)	139 (95.86)	145 (94.77)			213 (89.87)	80 (88.89)	71 (89.87)	62 (91.18)		
Yes	24 (3.95)	10 (3.24)	6 (4.14)	8 (5.23)			24 (10.13)	10 (11.11)	8 (10.13)	6 (8.82)		
Systemic and administration site reactions, n(%)					-	0.146					χ^2^ = 1.85	0.397
No	590 (97.20)	303 (98.06)	142 (97.93)	145 (94.77)			220 (92.83)	84 (93.33)	71 (89.87)	65 (95.59)		
Yes	17 (2.80)	6 (1.94)	3 (2.07)	8 (5.23)			17 (7.17)	6 (6.67)	8 (10.13)	3 (4.41)		
Ocular side effects, n(%)					-	1					-	1
No	606 (99.84)	308 (99.68)	145 (100.00)	153 (100.00)			236 (99.58)	89 (98.89)	79 (100.00)	68 (100.00)		
Yes	1 (0.16)	1 (0.32)	0 (0.00)	0 (0.00)			1 (0.42)	1 (1.11)	0 (0.00)	0 (0.00)		
Immune system side effects, n(%)					-	1					-	1
No	603 (99.34)	307 (99.35)	144 (99.31)	152 (99.35)			233 (98.31)	88 (97.78)	78 (98.73)	67 (98.53)		
Yes	4 (0.66)	2 (0.65)	1 (0.69)	1 (0.65)			4 (1.69)	2 (2.22)	1 (1.27)	1 (1.47)		

χ^2^, Chi-square test, −, Fisher exact.Bold values indicate statistical significance at *p* < 0.05.

## 4 Discussion

This study provides a comprehensive, platform-specific evaluation of the quality and reliability of semaglutide-related content on three predominant Chinese short video platforms. Our findings reveal significant disparities in content quality, user engagement, and the narrative framing of side effects across Bilibili, TikTok, and Rednote. These results underscore the profound influence of platform ecology and creator identity on the construction and dissemination of public health information.

The hierarchical gradient in content quality—Rednote > TikTok > Bilibili, as evidenced by both M-DISCERN and GQS scores—is a central finding of this work. This gradient can be interpreted through the distinct “affordances” and cultural norms of each platform (25–27). Rednote’s ecosystem, built upon in-depth, community-verified “notes” and a dominant user base actively seeking detailed lifestyle and health information (particularly regarding weight management), creates an environment that rewards evidence-based and well-contextualized content (4, 6). This “pro-knowledge” culture encourages creators to provide higher-quality information to gain credibility and traction. In contrast, TikTok’s core mechanic is rapid, algorithm-driven consumption for mass entertainment (28–30). While this allows health topics to achieve viral reach, the platform’s inherent “attention economy” often prioritizes sensationalism and brevity over depth and nuance, resulting in middling quality scores (31). Bilibili’s position at the bottom of the quality gradient is perhaps the most intriguing. Historically a hub for long-form, niche, and academically-oriented content, our data suggest that its foray into short-form video may have diluted this advantage for complex topics like pharmacology (3, 32, 33). The smaller initial audience for specialized medical content, combined with potential “information dilution” in the short-video format, may lead to a proliferation of low-effort, repetitive, or poorly-sourced videos, as low-quality content can easily fill a knowledge vacuum (34–36).

The critical role of the creator is further elucidated by our data. The significant superiority of professional individuals over non-professional individuals in both quality and engagement metrics highlights that expertise, when effectively communicated, is a key driver of reliable health communication (37, 38). However, the blurred line between professional and non-professional institutions—where significant differences were observed only in engagement metrics, not in core quality scores—warrants caution (39, 40). This suggests that institutional branding alone does not guarantee informational quality. It is plausible that some non-professional institutions (e.g., commercial media outlets) employ production values and narrative techniques that mimic authoritative sources, thereby achieving similar perceived quality scores without the underlying expertise, a phenomenon linked to the concept of “manufactured authenticity” (41, 42).

The high correlation between different engagement metrics (e.g., likes, shares, collects) indicates a cohesive user behavior pattern, where a video’s popularity is reinforced across multiple interactive dimensions (43). The absence of a correlation between publication time and engagement is a noteworthy insight for public health communicators (44). It suggests that for high-value, “evergreen” health topics like drug side effects, content quality and inherent utility are the primary determinants of long-term impact, not ephemeral algorithmic trends at the time of posting. This empowers creators to focus on substantive value rather than tactical timing (45).

Perhaps the most clinically significant finding lies in the “platformization” of side effect narratives. The statistically significant differences in the reporting of specific side effects (e.g., abnormal lab findings, gastrointestinal, and hepatobiliary effects) indicate that a user’s understanding of semaglutide’s risk profile is not based solely on clinical evidence, but is heavily mediated by the platform they use (6, 46). For instance, the emphasis on gastrointestinal side effects on certain platforms may reflect their relatability and potency in personal experience narratives, whereas more abstract “abnormal indicator” side effects might be highlighted on platforms with a more analytical or science-communication orientation (47, 48). This fragmentation of risk discourse can lead to an incomplete or biased public perception, where some risks are over-emphasized while others are minimized, depending on the digital silo one inhabits (4, 49). Our dual-lens analysis provides pivotal evidence for understanding how semaglutide side-effects are communicated on social media. In the full sample (*n* = 607), conventional statistics masked key differences; for example, no inter-platform disparity was detected for “abnormal laboratory findings”—a toxicity that requires specialist knowledge (*p* = 0.611). However, when the analytic lens was narrowed to the core sample of videos that actually mentioned adverse events (*n* = 237), the same comparison became significant (*p* = 0.036). This phenomenon stems from the fact that 61% of videos in the full cohort contained zero side-effect content, effectively diluting the risk signal. Only after excising this noise could genuine between-platform differences in highly specific, easily overlooked adverse events be detected. Importantly, the “platform effect” for gastrointestinal and hepatobiliary side-effects proved robust: regardless of whether the full or core sample was used, TikTok consistently displayed a higher prevalence of these intuitively perceptible adverse events than Bilibili or RedNote, indicating that the platform’s algorithm or audience preference amplifies the visibility of such salient toxicities. The dual-sample strategy offers four clear advantages. First, the core subset minimizes zero-event dilution, boosting statistical power to uncover subtle platform- or creator-level differences. Second, the approach marries a macroscopic trend (full sample) with microscopic validation (core sample), creating a “breadth-depth” double check that markedly strengthens inference. Third, findings directly inform precision public-health action by pinpointing which high-specialty risk topics (e.g., laboratory abnormalities) are systematically under-represented on which platforms, thereby supplying data-driven targets for platform-specific education or regulation. Finally, the framework is highly transferable: it can be replicated in studies of other drugs or health issues, preserving both population-level generalizability and granular detail while avoiding an “all-or-none” information loss.

These findings have several critical implications. For healthcare consumers and patients, our study serves as a cautionary note, urging critical appraisal of the platform source and creator credentials when seeking health information online. For health professionals and educators, there is a pressing need to actively engage with these platforms, particularly those like Rednote that demonstrate a receptivity to high-quality content, to ensure accurate voices are amplified (50). For content creators, the success of the “strong evidence + scenario-based” expression model on Rednote offers a valuable template for translating complex medical information into accessible yet reliable formats (51). From a policy perspective, our research highlights the need for platform-specific governance. A one-size-fits-all approach to health misinformation is unlikely to be effective. Platforms like TikTok, with their vast reach and entertainment focus, might require more robust fact-checking partnerships, whereas Bilibili could benefit from initiatives that bolster its nascent community of expert creators in the short-video domain (52).

This study is not without limitations. Firstly, its cross-sectional design provides a snapshot in time, and the dynamic nature of social media content necessitates longitudinal follow-up. Secondly, our quantitative analysis, while robust, could be enriched by qualitative methods, such as a discourse analysis of the comment sections, to understand user interpretation and concerns. Thirdly, the sample was limited to three major Chinese short-video platforms, which may constrain the generalizability of our findings to other platforms or cultural contexts. In particular, the study did not include international short-video platforms such as YouTube Shorts, due to substantial ethical, legal, and practical constraints in obtaining and analyzing health-related content across jurisdictions. Consequently, the conclusions should be interpreted primarily within the context of Chinese short-video ecosystems. Despite these limitations, the strengths of this study include a relatively large, systematically sampled dataset, the use of validated instruments (M-DISCERN and GQS) for quality assessment, and a novel comparative framework that explicitly links platform ecology to health information quality and specific risk narratives.

In conclusion, our investigation demonstrates that the landscape of semaglutide information on Chinese short video platforms is not a monolith but a varied ecosystem where quality and narrative are powerfully shaped by the interplay of platform design, creator expertise, and user engagement patterns. Recognizing this “platformized” reality is the first step toward developing more sophisticated, targeted, and effective strategies for digital health literacy and communication. Ensuring the reliable dissemination of pharmaceutical information in the digital age requires not only creating good content but also understanding the distinct soil in which it is meant to grow.

## 5 Conclusion

By analyzing 607 semaglutide-related short videos across three major Chinese platforms, this study revealed a consistent quality gradient of “Rednote > TikTok > Bilibili”, underscoring the significant role of platform ecology in shaping information dissemination. Side-effect narratives exhibited a distinct “platformization” pattern, with significant differences in the mention of gastrointestinal and hepatobiliary adverse events across platforms. The dual-perspective analysis further demonstrated that such disparities were more pronounced in the side effect-focused subsample, highlighting how platforms selectively filter risk information. These findings indicate that the drug information accessible to the public is structurally biased. Future interventions must therefore be tailored to platform-specific characteristics to enhance the reliability and completeness of online pharmaceutical content.

## Data Availability

The original contributions presented in the study are included in the article/Supplementary material, further inquiries can be directed to the corresponding author.
