# Supplementary material for: Quality and reliability of semaglutide content on different Chinese short video platforms: a study focusing on side effects
Source: Front Public Health. 2026 Feb 24;14:1750738. doi: 10.3389/fpubh.2026.1750738 (PMC12971943; doi:10.3389/fpubh.2026.1750738)
Supplement: Supplementary file 2 [file Table_2.docx]

**Supplementary Table 1. Summary of Adverse Events for Semaglutide Tablets.**

**International Nonproprietary Name (INN):** Semaglutide Tablets
**Brand Names:** Rybelsus (global); 诺和忻 (China)
**Product Information Source:** <https://db.yaozh.com/instruct/3720831349579136.html>

Side effects of Semeglutide Tablets

| **System Organ Classification** | **Very common** | **common** | **Occasionally** | **rare** | **unknown** |
| --- | --- | --- | --- | --- | --- |
| immune system disease |  |  | Hypersensitive reaction ^c^ | Immediate severe allergic reaction |  |
| Metabolic and nutritional diseases | Hypoglycemia ^a^ ( combined with insulin or sulfonylureas ) | Hypoglycemia ^a^ ( combined with other oral antidiabetic drugs ) Loss of appetite |  |  |  |
| Eye organ diseases |  | Diabetic retinopathy complications ^b^ |  |  |  |
| Heart organ disease |  |  | Increased heart rate |  |  |
| Gastrointestinal system diseases | Nausea, diarrhea | Vomiting , Abdominal pain, Abdominal distension，Constipation，Indigestion | Belching  Delayed gastric emptying | acute pancreatitis | Intestinal obstruction ^d^ |
|  |  | Gastritis，Gastrointestinal bloating |  |  |  |
|  |  | Gastroesophageal reflux disease |  |  |  |
| hepatobiliary system disease |  |  | cholelithiasis |  |  |
| Systemic diseases and various reactions at the site of administration |  | fatigue |  |  |  |
| Various inspections |  | Elevated lipase，Elevated amylase | weight loss |  |  |
| Nervous system diseases |  | dizziness | parageusis |  |  |

a. Hypoglycaemia was defined as a plasma glucose level <3.0 mmol/L (<54 mg/dL).

b. The composite endpoint “diabetic retinopathy complications” comprised retinal photocoagulation, intravitreal pharmacotherapy, vitreous haemorrhage, and diabetes-related blindness (rare). The incidence is derived from cardiovascular-outcome trials of subcutaneous semaglutide; the same risk cannot be excluded for the oral formulation.

c. Based on post-marketing adverse-drug-reaction data; includes grouped terms for hypersensitivity-related events (e.g., rash and urticaria).

d. Based on post-marketing adverse-drug-reaction reports.

**Supplementary Table 2. Summary of Adverse Events for Semaglutide Injection.**

**International Nonproprietary Name (INN):** Semaglutide Injection
**Brand Names:** Ozempic (global); 诺和泰 (China)
**Product Information Source:** <https://ypk.39.net/2310026/manual/>

Adverse Effects of Semaglutide Injection

Summary of Safety Profile

A total of 4792 patients were exposed to semaglutide across 8 Phase IIa trials. The most commonly reported adverse reactions (ARs) in clinical trials were gastrointestinal disorders, including nausea (very common), diarrhea (very common), and vomiting (common). Typically, these reactions were mild or moderate in severity and of short duration.

The frequency of ARs was derived from pooled data of Phase IIa trials (excluding cardiovascular outcome trials) and is presented by system organ class and absolute frequency. Frequency definitions are as follows: very common (≥1/10); common (≥1/100 to <1/10); uncommon (≥1/1000 to <1/100); rare (≥1/10000 to <1/1000); and very rare (<1/10000). Within each frequency category, ARs are listed in decreasing order of severity.

2-Year Cardiovascular Outcome and Safety Trial

In populations at high cardiovascular risk, the safety profile of semaglutide was consistent with the ARs observed in other Phase IIa trials.

**Description of Selected Adverse Reactions**

**·Hypoglycemia:** No severe hypoglycemic events were observed with semaglutide monotherapy. Severe hypoglycemia was primarily reported when semaglutide was coadministered with sulfonylureas (1.2% of subjects, 0.03 events per patient-year) or insulin (1.5% of subjects, 0.02 events per patient-year). Very few hypoglycemic events were observed when semaglutide was combined with oral antidiabetic drugs other than sulfonylureas (0.1% of subjects, 0.001 events per patient-year).

**·Gastrointestinal Adverse Reactions:** Nausea occurred in 17.0% and 19.9% of patients in the semaglutide 0.5 mg and 1 mg groups, respectively; diarrhea in 12.2% and 13.3%; and vomiting in 6.4% and 8.4%. Most events were mild to moderate in severity and short-lived. Treatment discontinuation due to adverse reactions occurred in 3.9% and 5.0% of patients in the 0.5 mg and 1 mg groups, respectively. These events were most frequently reported during the first few months of treatment. Patients with low body weight may experience more gastrointestinal side effects when treated with semaglutide.

**·Acute Pancreatitis:** In Phase IIa trials, the incidence of adjudicated acute pancreatitis was 0.3% in the semaglutide group and 0.2% in the control group. In the 2-year cardiovascular outcome trial, the incidence of adjudicated acute pancreatitis was 0.5% in the semaglutide group and 0.6% in the placebo group.

**·Diabetic Retinopathy Complications:** A 2-year clinical trial enrolled 3,297 patients with type 2 diabetes who were at high cardiovascular risk, had a long duration of diabetes, and poor glycemic control. In this trial, a higher proportion of patients in the semaglutide group (3.0%) experienced adjudicated diabetic retinopathy complications compared with the placebo group (1.8%). This finding was also observed in patients with pre-existing diabetic retinopathy who received insulin treatment. The between-group difference emerged early in the treatment and persisted throughout the trial. A systematic assessment of diabetic retinopathy complications was only conducted in cardiovascular outcome trials. In a 1-year clinical trial involving 4,807 patients with type 2 diabetes, the incidence of diabetic retinopathy-related adverse events was similar between the semaglutide group (1.7%) and the active comparator group (2.0%).

**·Treatment Discontinuation Due to Adverse Events:** The incidence of treatment discontinuation due to adverse events was 6.1% and 8.7% in patients receiving semaglutide 0.5 mg and 1 mg, respectively, compared with 1.5% in the placebo group. Gastrointestinal events were the most common reason for discontinuing semaglutide.

**·Injection Site Reactions:** Injection site reactions (e.g., rash, erythema at the injection site) were reported in 0.6% and 0.5% of patients treated with semaglutide 0.5 mg and 1 mg, respectively. These reactions were generally mild in severity.

**·Immunogenicity:** Consistent with the potential immunological properties of protein- or peptide-containing medications, patients may develop antibodies following treatment with semaglutide. The proportion of patients testing positive for anti-semaglutide antibodies at any time after baseline was low (1–2%). No patients developed anti-semaglutide neutralizing antibodies or antibodies with neutralizing effects on endogenous GLP-1 by the end of the trial.

**·Increased Heart Rate:** Increases in heart rate have been reported with the use of GLP-1 receptor agonists. Among subjects treated with semaglutide in Phase IIa trials, a mean increase in heart rate of 1 to 6 beats per minute (bpm) from baseline (72–76 bpm) was observed. In a long-term trial involving subjects with cardiovascular risk, 16% of semaglutide-treated subjects had an increase in heart rate of ≥10 bpm after 2 years of treatment, compared with 11% in the placebo group.

**·Reporting of Suspected Adverse Reactions:** Reporting of suspected adverse reactions after the medicinal product is authorized is important to enable continuous monitoring of the benefit/risk balance of the medicinal product. Healthcare professionals should report any suspected adverse reactions through the national reporting system.

**· Contraindications:** Hypersensitivity to the active substance of semaglutide or any of its excipients. Personal or family history of medullary thyroid carcinoma (MTC), or patients with multiple endocrine neoplasia syndrome type 2 (MEN 2) .

[**Supplementary Table 3**](#_Toc27045)**. Global Quality Score (GQS) (Scoring ranges from 1 to 5)**

| **Reliability Score** | **Score** |
| --- | --- |
| Is the video clear, concise, and understandable? | 1 |
| Are valid sources cited? | 2 |
| Is the content presented balanced and unbiased? | 3 |
| Are additional sources of content listed for patient reference? | 4 |
| Are areas of uncertainty mentioned? | 5 |

[**Supplementary Table 4**](#_Toc27045)**. Modified DISCERN quality criteria for assessing the reliability of video. (1 point for answer ‘yes’, 0 point for answer ‘no’)**

| **DISCERN Definition** | **Score** |
| --- | --- |
| Poor quality：Specifically, the content is illogical, the mobility is poor, most of the information is missing, and it is useless for patients. | 1 |
| Generally poor quality ：the content logic is poor, although some information is listed, more important information is still missing, and the use of patients is very limited. | 2 |
| Moderate quality：some important information is adequately discussed. | 3 |
| Good quality and flow：Specifically, the video logic is clear and smooth, covering most of the relevant information, which is useful for patients. | 4 |
| Excellent quality and flow：Specifically, the video logic is clear, and the content is very smooth, which is very useful for patients. | 5 |

[**Supplementary Table 5**](#_Toc27045)**. Characteristics, Quality, and Reliability of Semaglutide-Related Videos by Different Individual Uploaders on Different Chinese Short-Video Platforms.**

| Variables | Total (n = 541) | Non-professional individuals (n = 242) | specialized individual (n = 299) | Statistic | *P* |
| --- | --- | --- | --- | --- | --- |
|  |  |  |  |  |  |
| M Discern Score, Mean ± SD | 1.53 ± 0.85 | 1.21 ± 0.77 | 1.80 ± 0.81 | t=-8.68 | **<.001** |
| GQS score, Mean ± SD | 2.20 ± 0.91 | 1.78 ± 0.77 | 2.55 ± 0.87 | t=-10.93 | **<.001** |
| Duration, M (Q₁, Q₃) | 124.00 (68.00, 208.00) | 148.50 (99.00, 276.25) | 110.00 (56.00, 158.00) | Z=-5.33 | **<.001** |
| Like, M (Q₁, Q₃) | 45.00 (9.00, 566.00) | 14.50 (4.00, 71.00) | 144.00 (22.00, 1778.00) | Z=-8.75 | **<.001** |
| Comment, M (Q₁, Q₃) | 17.00 (2.00, 140.00) | 10.00 (1.25, 58.75) | 25.00 (2.50, 282.00) | Z=-3.33 | **<.001** |
| Share, M (Q₁, Q₃) | 15.00 (2.00, 347.00) | 4.00 (0.00, 33.50) | 70.00 (6.50, 1259.50) | Z=-9.19 | **<.001** |
| Collect, M (Q₁, Q₃) | 20.00 (3.00, 229.00) | 6.00 (1.00, 31.00) | 53.00 (7.00, 805.50) | Z=-8.76 | **<.001** |

t: t-test, Z: Mann-Whitney test SD: standard deviation, M: Median, Q₁: 1st Quartile, Q₃: 3st Quartile

[**Supplementary Table 6**](#_Toc27045)**. Characteristics, Quality, and Reliability of Semaglutide-Related Videos by Different Institutional Uploaders on Different Chinese Short-Video Platforms.**

| Variables | Total (n = 66) | Non-professional institutions (n = 27) | professional institutions (n = 39) | Statistic | *P* |
| --- | --- | --- | --- | --- | --- |
|  |  |  |  |  |  |
| M Discern Score, Mean ± SD | 1.64 ± 0.94 | 1.37 ± 0.93 | 1.82 ± 0.91 | t=-1.96 | 0.055 |
| GQS score, Mean ± SD | 2.36 ± 0.97 | 2.63 ± 0.93 | 2.18 ± 0.97 | t=1.89 | 0.064 |
| Duration, M (Q₁, Q₃) | 127.00 (52.50, 241.00) | 102.00 (40.50, 217.00) | 148.00 (60.00, 310.50) | Z=-1.45 | 0.146 |
| Like, M (Q₁, Q₃) | 30.00 (7.25, 519.25) | 360.00 (25.00, 2192.00) | 14.00 (4.00, 94.50) | Z=-3.12 | **0.002** |
| Comment, M (Q₁, Q₃) | 16.50 (1.00, 224.75) | 238.00 (6.00, 656.50) | 6.00 (0.50, 46.00) | Z=-2.71 | **0.007** |
| Share, M (Q₁, Q₃) | 12.50 (3.25, 232.50) | 72.00 (6.50, 3293.00) | 7.00 (2.00, 69.50) | Z=-2.43 | **0.015** |
| Collect, M (Q₁, Q₃) | 15.50 (4.00, 165.75) | 127.00 (4.50, 378.00) | 11.00 (4.00, 38.00) | Z=-0.98 | 0.328 |
| t: t-test, Z: Mann-Whitney test SD: standard deviation, M: Median, Q₁: 1st Quartile, Q₃: 3st Quartile | | | | | |
|  | | | | | |

[**Supplementary Table 7**](#_Toc27045)**. Presentation and Comparison of Video Content in Videos on Different Chinese Short-Video Platforms.**

| Variables | Total (n = 607) | bilibili (n = 309) | Rednote (n = 145) | TikTok (n = 153) | Statistic | *P* |
| --- | --- | --- | --- | --- | --- | --- |
|  |  |  |  |  |  |  |
| Drug Information / Medication Education, n(%) |  |  |  |  | χ²=18.07 | **<.001** |
| no | 309 (50.91) | 179 (57.93) | 53 (36.55) | 77 (50.33) |  |  |
| yes | 298 (49.09) | 130 (42.07) | 92 (63.45) | 76 (49.67) |  |  |
| Policy & News, n(%) |  |  |  |  | χ²=15.27 | **<.001** |
| no | 511 (84.18) | 249 (80.58) | 137 (94.48) | 125 (81.70) |  |  |
| yes | 96 (15.82) | 60 (19.42) | 8 (5.52) | 28 (18.30) |  |  |
| Clinical Guidance / Medication Instructions, n(%) |  |  |  |  | χ²=98.54 | **<.001** |
| no | 398 (65.57) | 255 (82.52) | 52 (35.86) | 91 (59.48) |  |  |
| yes | 209 (34.43) | 54 (17.48) | 93 (64.14) | 62 (40.52) |  |  |
| Patient Experiences / Usage Sharing, n(%) |  |  |  |  | χ²=55.84 | **<.001** |
| no | 439 (72.32) | 187 (60.52) | 136 (93.79) | 116 (75.82) |  |  |
| yes | 168 (27.68) | 122 (39.48) | 9 (6.21) | 37 (24.18) |  |  |
| Promotional Content, n(%) |  |  |  |  | χ²=36.58 | **<.001** |
| no | 488 (80.40) | 219 (70.87) | 133 (91.72) | 136 (88.89) |  |  |
| yes | 119 (19.60) | 90 (29.13) | 12 (8.28) | 17 (11.11) |  |  |

χ²: Chi-square test
